# Supplementary material for: Promotion of homology-directed DNA repair by polyamines
Source: Nat Commun. 2019 Jan 8;10:65. doi: 10.1038/s41467-018-08011-1 (PMC6325121; doi:10.1038/s41467-018-08011-1)
Supplement: Supplementary file 1 — Supplementary Information [file 41467_2018_8011_MOESM1_ESM.pdf]

Supplementary Information

Lee et al, Promotion of homology-directed DNA repair by polyamines

This PDF file includes:

Supplementary Figures 1 to 14

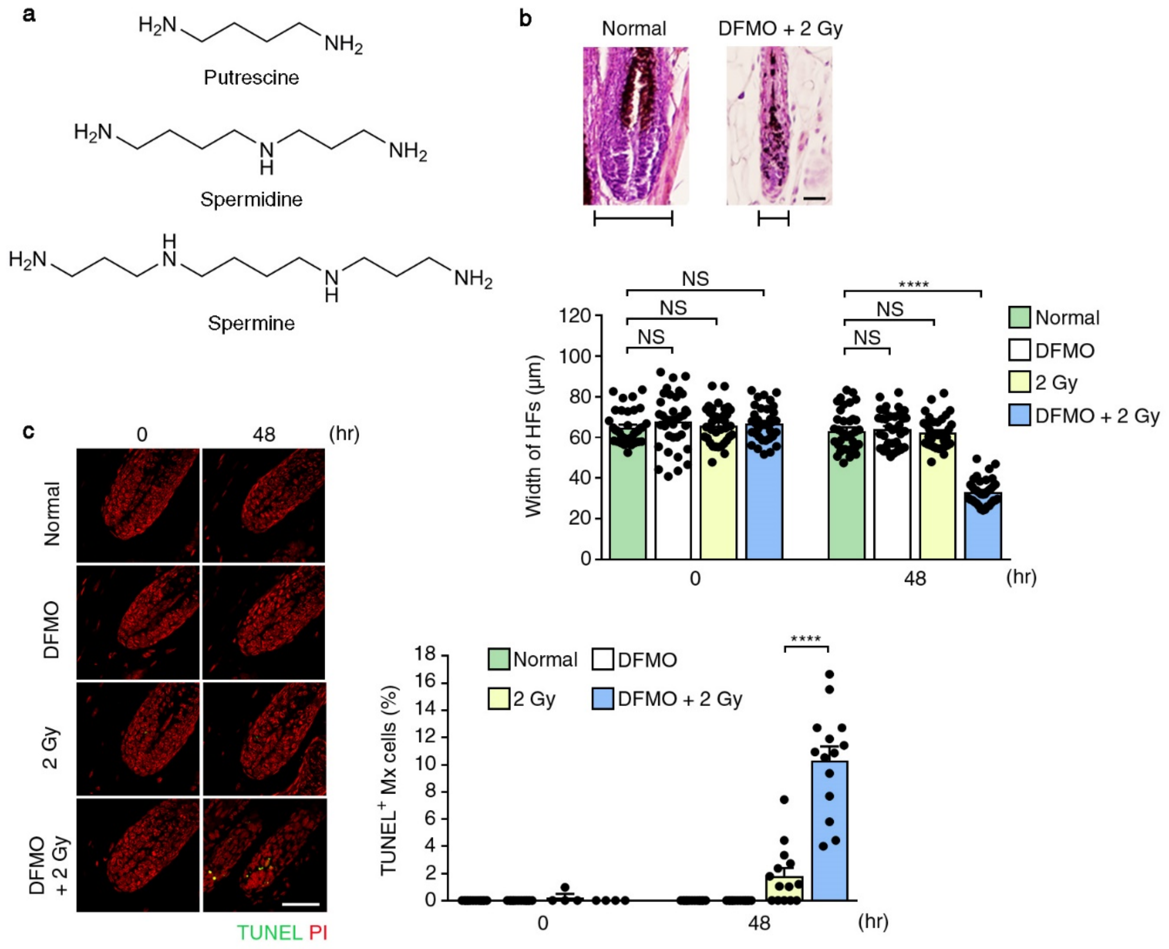

Supplementary Fig. 1 Polyamine depletion sensitizes hair follicles to radiation damage.
a Structures of the major cellular polyamines: putrescine, spermidine, and spermine.
b Histology of murine hair follicles after treatment with DFMO, 2 Gy ionizing radiation, or with both DFMO and radiation. Representative pictures of hair bulb without or with DFMO and ionizing radiation treatment are shown. The width of hair follicles (HFs) was measured at the indicated time points. (n = 36 hair follicles). Scale bar, 30 μm.
c Representative pictures of TUNEL staining (left) and the percentages of TUNEL-positive matrix cells (right) with different treatments are shown. Propidium iodide (PI) is used as a DNA stain. (n = 14 hair follicles). Scale bar, 50 μm.
****$P < 0.0001$; NS, not significant ($P > 0.05$). Data are the mean ± s.e.m. from three independent experiments. Statistics was performed by one-way ANOVA with Tukey's post hoc test. Raw data are provided as a Source Data file.

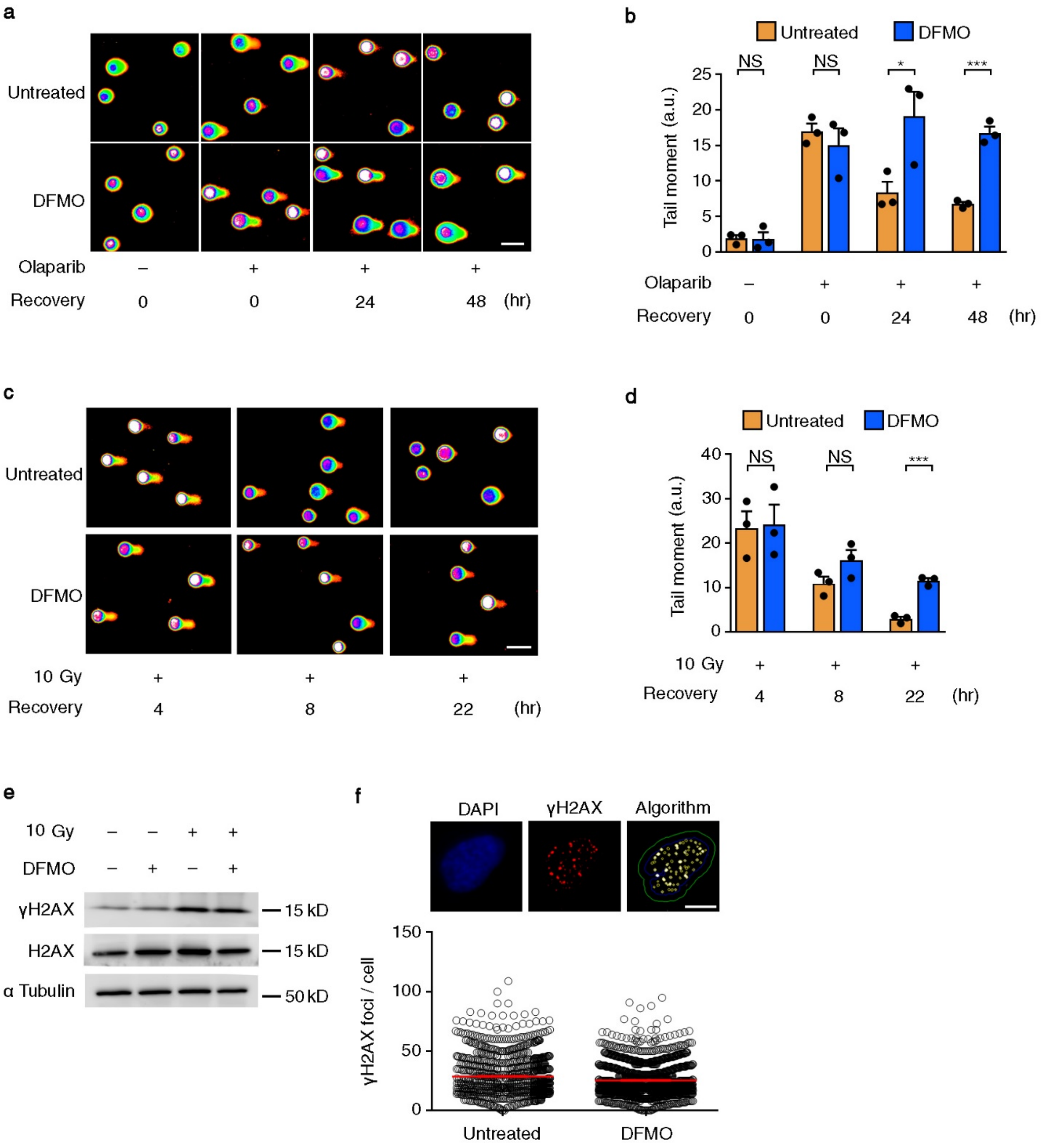

Supplementary Fig. 2 Depletion of polyamines compromises DNA damage repair.

a, b Assessment of the level of DNA damage by the comet assay. DFMO-pretreated or untreated cells were incubated with 125 nM olaparib for 24 hours and analyzed at the indicated recovery times. Representative images are shown in **a** and the tail moment analysis of DNA damage is shown in **b**. Each of the three independent experiments measured the mean of tail moment from fifty randomly selected cells. a.u., arbitrary units. Scale bar, 100 µm.

c-f DFMO-pretreated or untreated cells were exposed to ionizing radiation and analyzed at the indicated recovery times. Representative images are shown in **c** and the tail moment analysis of DNA damage is shown in **d**. Each of the three independent experiments measured the mean of tail moment from fifty randomly selected cells. a.u., arbitrary units. Scale bar, 100 µm. **e** Cell lysates at 4 hours post-irradiation were subjected to immunoblot analysis. **f** Representative images of γH2AX foci 4 hours after radiation exposure. The upper-right panel shows automated algorithm used to count γH2AX foci. Each circle represents the number of γH2AX foci from a single cell. The mean ± s.e.m. is indicated by the red line. n = 1675 for untreated control cells or n = 1336 for DFMO-pretreated cells from three independent experiments. Scale bar, 15 µm.

*$P < 0.05$; ***$P < 0.001$; NS, not significant ($P > 0.05$). Data are the mean ± s.e.m. from three independent experiments (n = 3). Statistics was performed by unpaired two-tailed Student's t-test. Raw data are provided as a Source Data file.

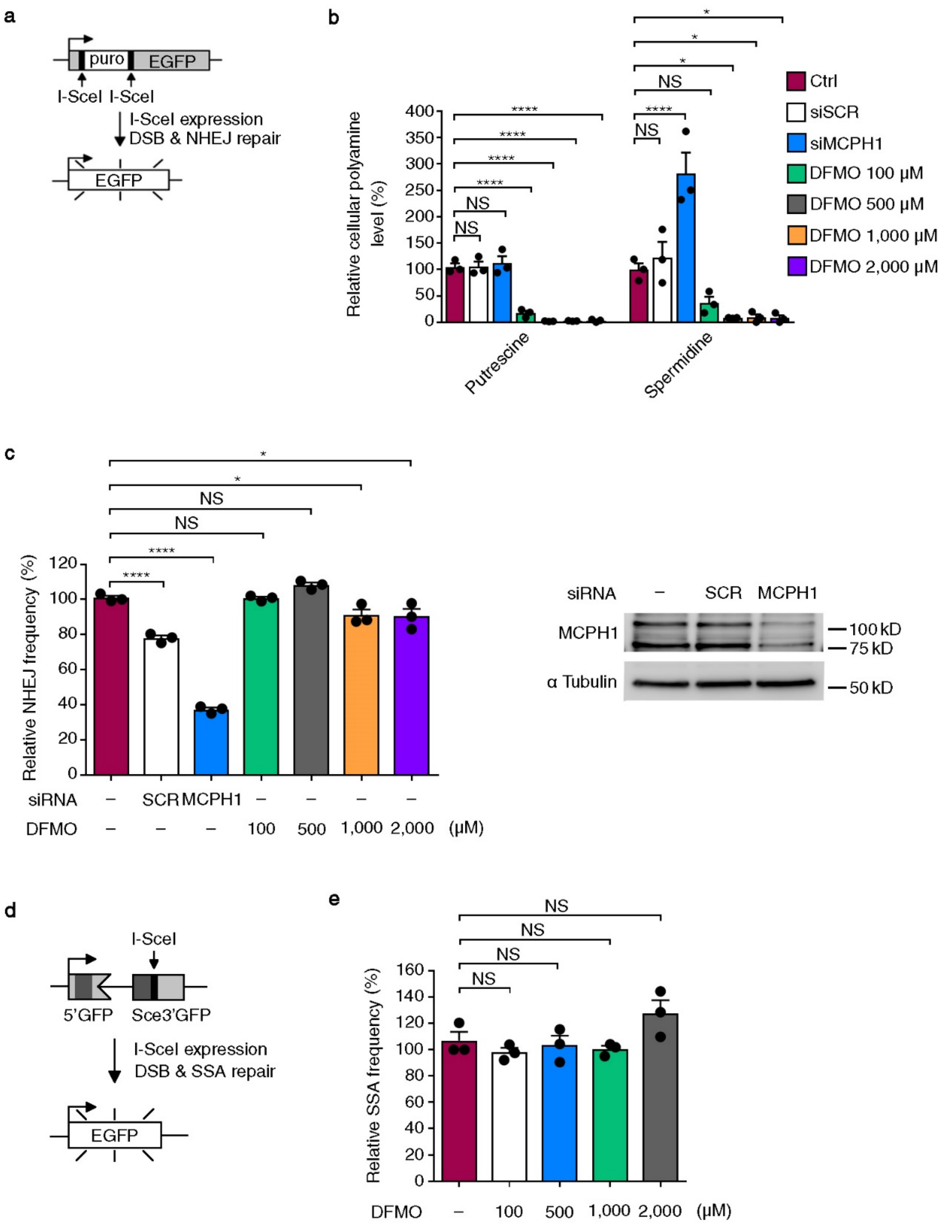

Supplementary Fig. 3 Polyamine depletion has no significant impact on non-homologous end-joining and single-strand annealing.

a Schematic of the EJ5-GFP reporter assay to assess NHEJ proficiency.

b, **c** U2OS EJ5-GFP cells were treated with the indicated amount of DFMO for 24 hours or transfected with the indicated siRNA for 48 hours. The level of individual polyamine was quantified and normalized to that in untreated cells (Ctrl) in **b**, and GFP^+ cells were quantified by flow cytometry in **c** after 72 hours of I-SceI transfection. The percentage of GFP^+ cells which represented relative NHEJ activity was normalized to untreated cells. MCPH1 was included as a positive control. Both MCPH1 isoforms were knocked down. Note that EJ5-GFP cells do not possess a significant amount of spermine to be detected. Expression levels of MCPH1 and tubulin were revealed by immunoblot analysis. SCR, Scrambled RNA.

d Schematic of the SA-GFP reporter assay to assess SSA activity.

e HEK293 cells were transfected with SA-GFP reporter plasmid for 48 hours, followed by treatment with indicated amount of DFMO for 24 hours. Following I-SceI transfection for 72 hours, GFP^+ cells were quantified by flow cytometry. The percentage of GFP^+ cells was normalized to untreated cells and represented as relative SSA frequency.
$^*P < 0.05$; $^{****}P < 0.0001$; NS, not significant ($P > 0.05$). Data are the mean ± s.e.m. from three independent experiments (n = 3). Statistics was performed by one-way ANOVA with Tukey's post hoc test. Raw data are provided as a Source Data file.

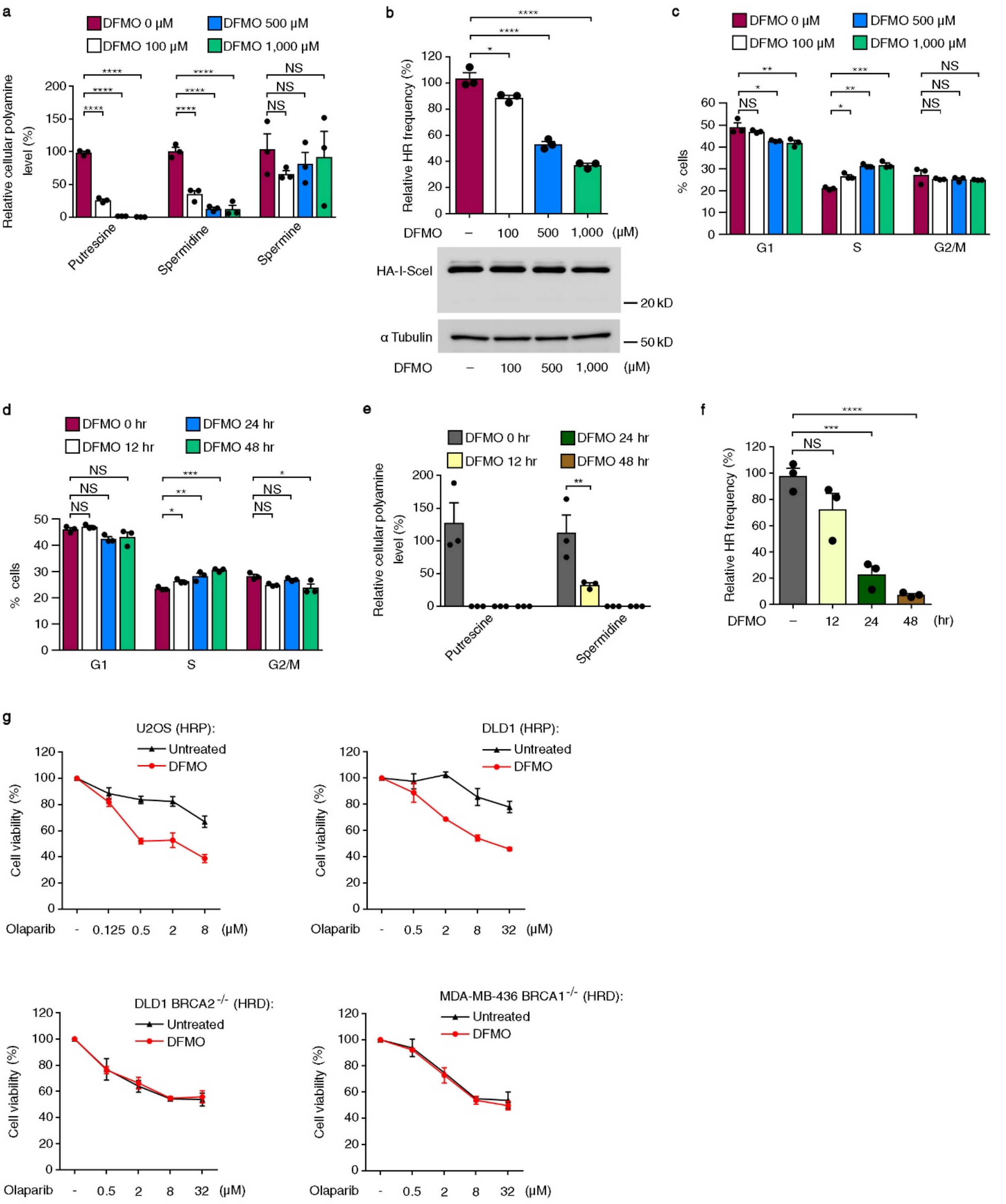

Supplementary Fig. 4 Attenuation of homologous recombination by depletion of polyamines.
a-c U2OS DR-GFP cells were treated with the indicated concentrations of DFMO for 24 hours. The level of individual polyamine was quantified and normalized to untreated cells in **a**, and GFP^+ cells representative of HR activity were quantified by flow cytometry in **b** after 48 hours of I-SceI transfection. The percentage of GFP^+ cells was normalized to untreated cells and represented as relative HR frequency. Expression levels of I-SceI and tubulin were revealed by immunoblot analysis. **c** The cell cycle profile of DFMO-treated cells was determined.
d Cell cycle profiling of 500 μM DFMO-treated U2OS cells for the indicated times.
e, **f** HEK293 cells were treated with 300 μM DFMO for the indicated times. The level of individual polyamine was quantified and normalized to untreated cells in **e**. Note that HEK293 DR-GFP cells

do not possess a significant amount of spermine to be detected. The HR efficiency was determined using DR-GFP reporter assay in **f**.

g Viability of cells pretreated with DFMO or not was measured after treatment with olaparib. U2OS, DLD-1 BRCA2$^{-/-}$, and MDA-MB-436 cells were treated with 200 μM DFMO. Significant response of DLD-1 wild-type cells to olaparib was treated with 300 μM DFMO. HRP, HR-proficient; and HRD, HR-deficient.

*$P < 0.05$; **$P < 0.01$; ***$P < 0.001$; ****$P < 0.0001$; NS, not significant ($P > 0.05$). Data are the mean ± s.e.m. from three independent experiments (n = 3). Statistics was performed by one-way ANOVA with Tukey's post hoc test. Raw data are provided as a Source Data file.

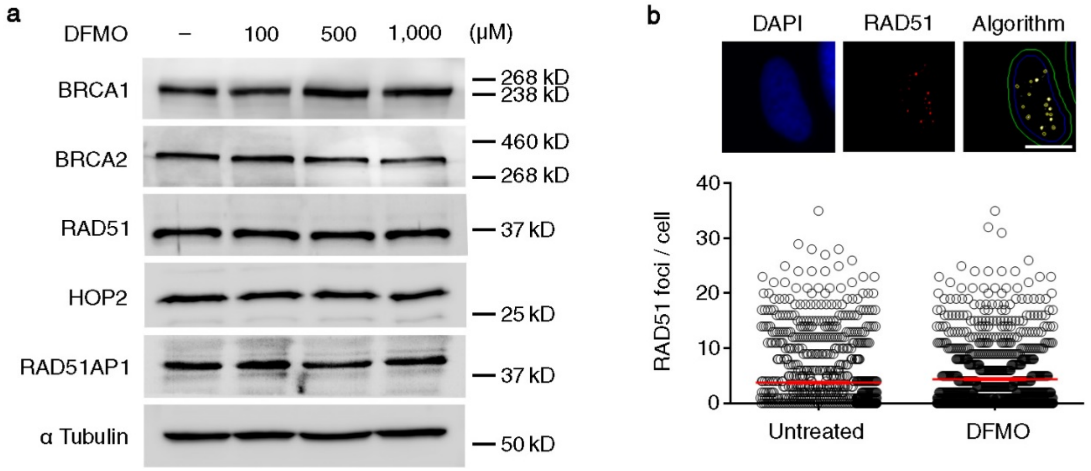

Supplementary Fig. 5 Polyamine depletion does not affect HR protein expression or DNA damage induced RAD51 focus formation.

a U2OS cells were treated with the indicated concentrations of DFMO for three days. Cell lysates were subjected to immunoblot analysis to evaluate expression levels of HR proteins as indicated.

b U2OS cells were pretreated with 500 μM DFMO for three days and then exposed to 10 Gy ionizing radiation. Representative images of RAD51 foci 4 hours after radiation exposure are shown. The upper-right panel shows the automated fluorescence microscopy algorithm used to count RAD51 foci. Each circle represents the number of RAD51 foci from a single cell. The mean ± s.e.m. from three independent experiments is indicated by the red line. n = 1374 for untreated control cells or n = 1058 for DFMO-pretreated cells from three independent experiments. Scale bar, 15 μm. Raw data are provided as a Source Data file.

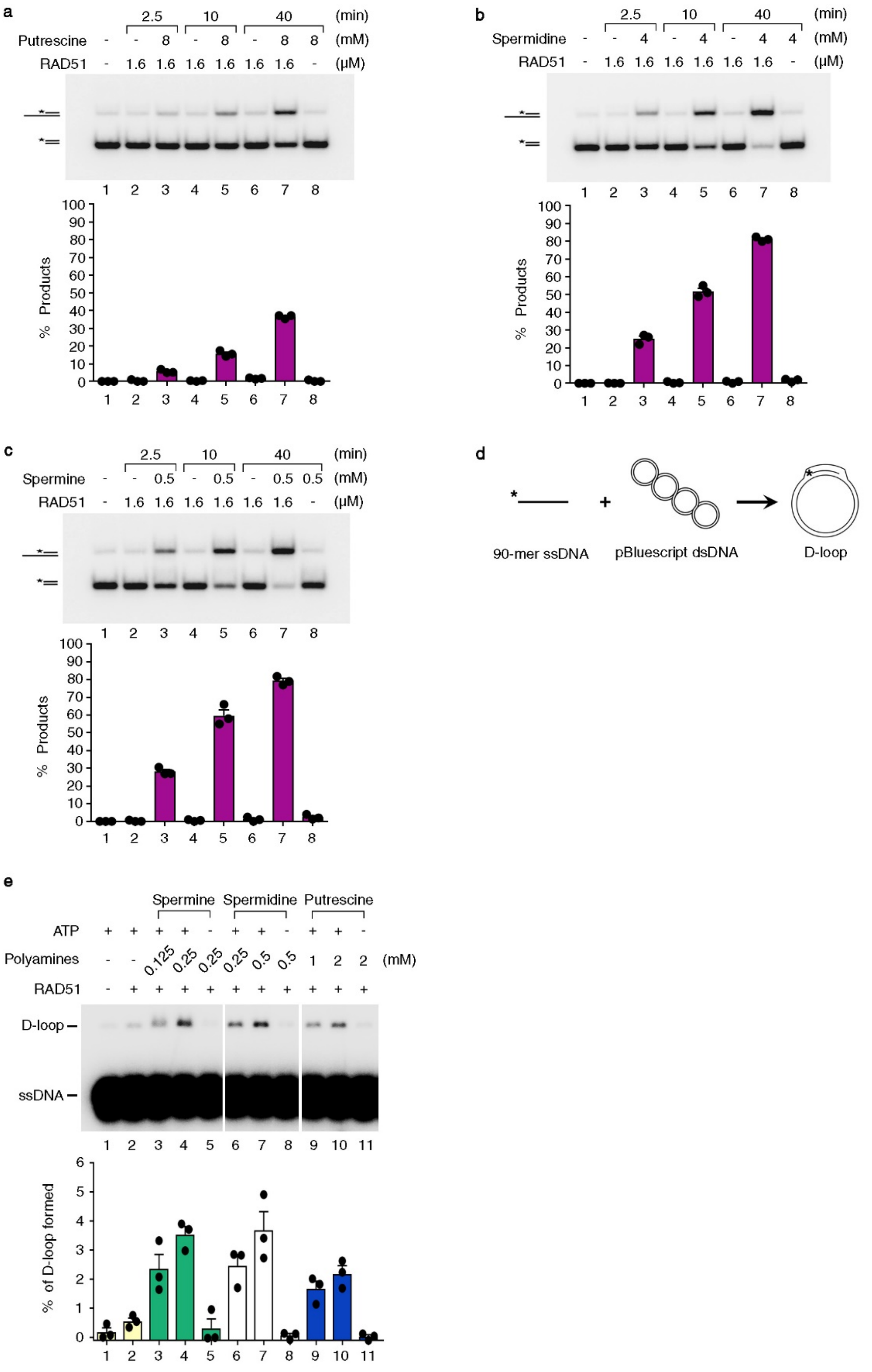

Supplementary Fig. 6 Polyamines stimulate RAD51 recombinase activity.

a-c Putrescine (**a**), spermidine (**b**) or spermine (**c**) enhances the activity of RAD51-mediated DNA strand exchange in a time-dependent manner.

d Schematic of the D-loop assay. The asterisk denotes the ^{32}P-label.

e D-loop formation was examined with the indicated concentration of putrescine, spermidine, or spermine.

a, b, c, e To normalize the percentage of product, each signal was subtracted from the percentage of product in control reaction (lane 1). Data are the mean ± s.e.m. from three independent experiments (n = 3). Raw data are provided as a Source Data file.

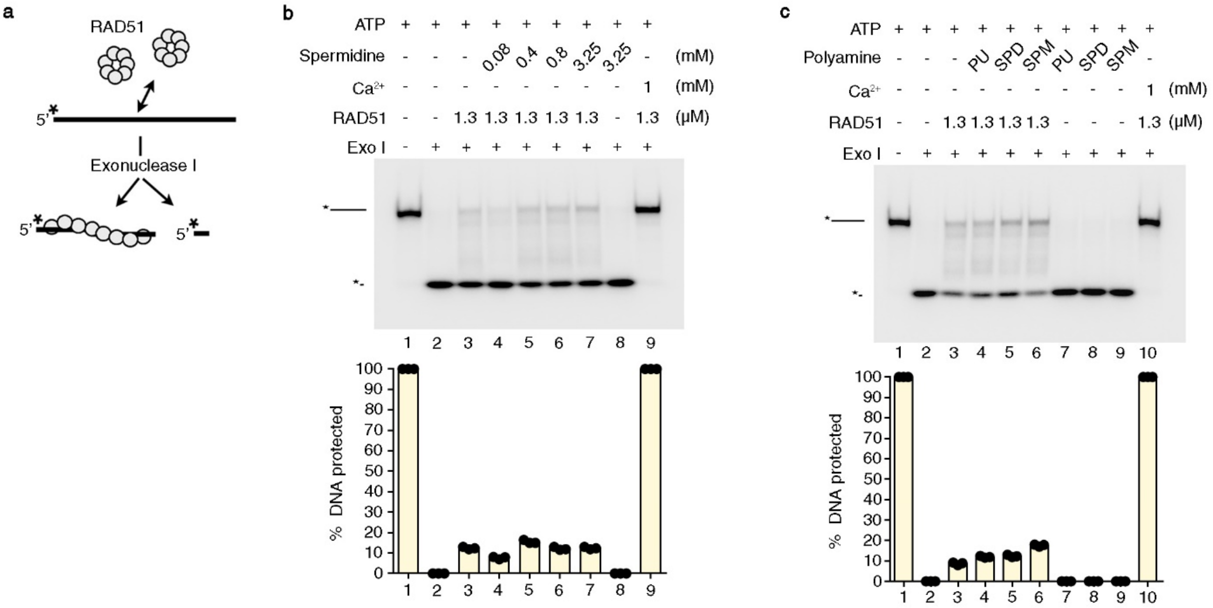

Supplementary Fig. 7 Polyamines do not enhance the stability of the RAD51 presynaptic filament.

a Schematic of the exonuclease I protection assay. Briefly, RAD51 presynaptic filament assembled on 5' ^{32}P-labeled ssDNA is treated with exonuclease I to test the stability of the presynaptic filament. The asterisk denotes the ^{32}P label.

b The RAD51 presynaptic filament was treated with exonuclease I in the absence or presence of the indicated concentrations of spermidine.

c Exonuclease I treatment was conducted in the absence or presence of putrescine (PU, 8 mM), spermidine (SPD, 4 mM), or spermine (SPM, 0.5 mM).

b, **c** Note that the inclusion of Ca^{2+} led to a strong stabilization of the presynaptic filament[20].

Data are the mean ± s.e.m. from three independent experiments (n = 3). Raw data are provided as a Source Data file.

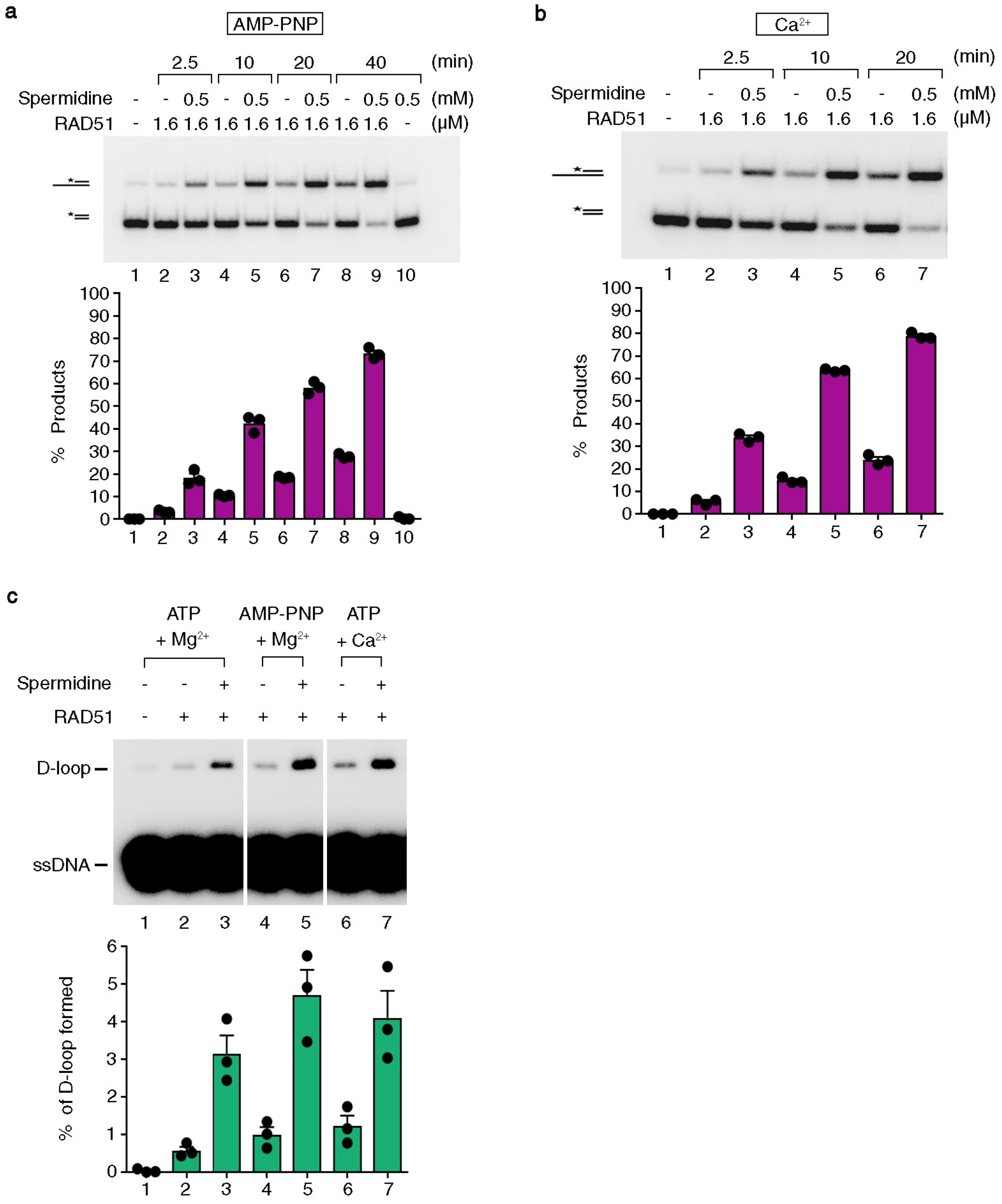

Supplementary Fig. 8 Spermidine enhances RAD51-mediated DNA strand exchange in the presence of AMP-PNP and Ca^{2+}.

a, **b** DNA strand exchange was examined with spermidine for the indicated time point. The presynaptic filament was assembled using 0.25 mM Mg^{2+}-1 mM AMP-PMP (**a**) or 0.5 mM Ca^{2+}-1 mM ATP (**b**) as the cofactor. The asterisk denotes the ^{32}P label.

c Spermidine stimulates D-loop formation of the stable presynaptic filament which was assembled using 1 mM AMP-PMP or 0.5 mM Ca^{2+} as the cofactor.

To normalize the percentage of product, each signal was subtracted from the percentage of product in control reaction (lane 1). Data are the mean ± s.e.m. from three independent experiments (n = 3). Raw data are provided as a Source Data file.

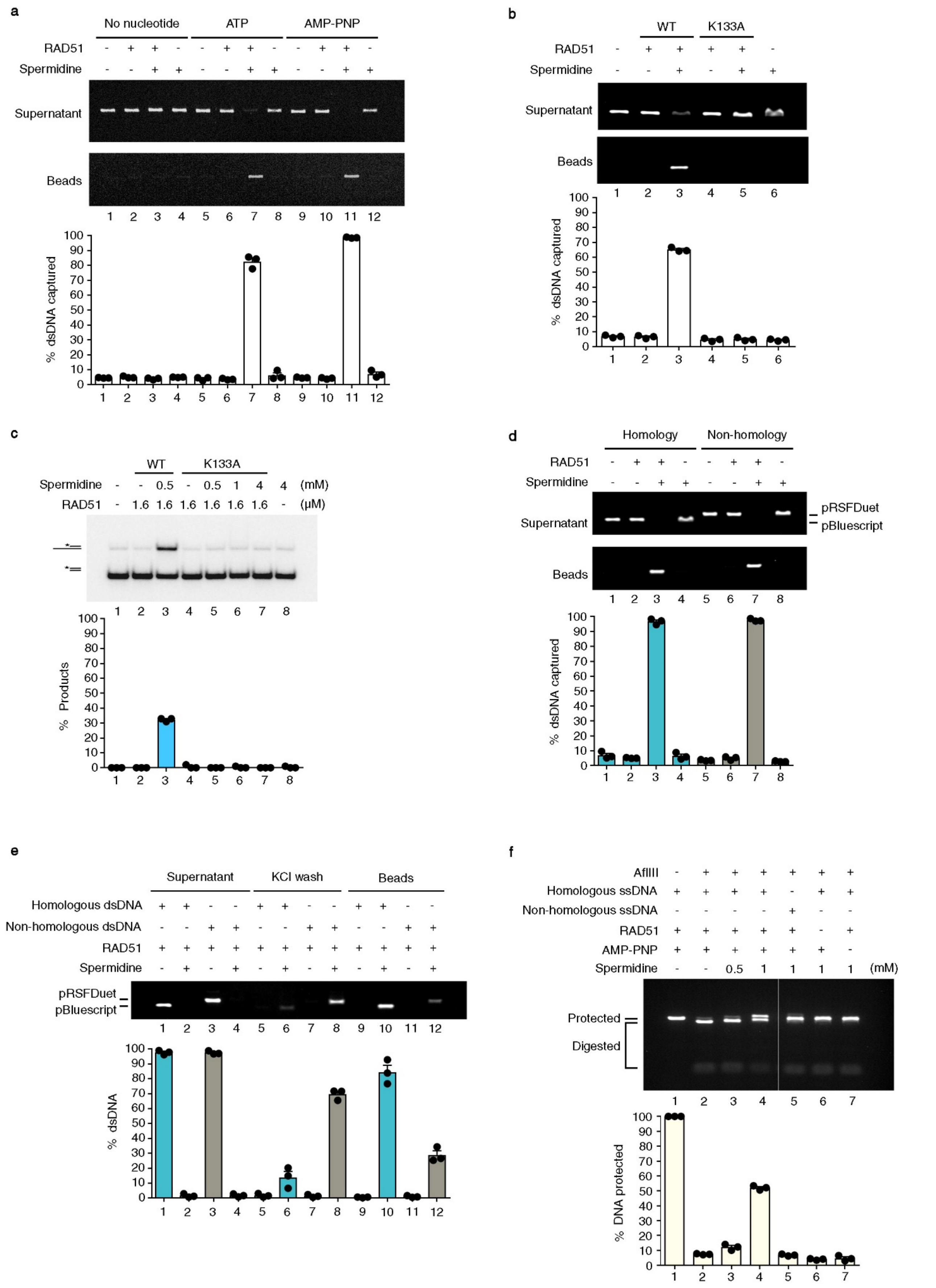

Supplementary Fig. 9 Effect of spermidine on synaptic complex assembly.

a The presynaptic filament was assembled with ATP or AMP-PNP as the cofactor and its ability to capture non-homologous duplex DNA was examined with or without spermidine (4 mM) being

present. As control, RAD51 was examined for duplex capture in the absence of a nucleotide.
b RAD51 (WT) and RAD51 K133A were examined for duplex capture activity without or with spermidine (2 mM) being present. ATP was used as the nucleotide cofactor.
c RAD51 and RAD51 K133A were examined for DNA strand activity without or with spermidine. ATP was used as the nucleotide cofactor. The reaction time was 20 min. The asterisk denotes the ^{32}P-label.
d Capture of homologous (pBluescript) or non-homologous (pRSFDuet) duplex DNA by the RAD51 presynaptic filament was examined without or with spermidine (4 mM). AMP-PNP was used as the nucleotide cofactor.
e Capture of homologous (pBluescript) or non-homologous (pRSFDuet) duplex DNA by the RAD51 presynaptic filament was examined as in **d**. The stability of the ternary complex comprising the presynaptic filament and the captured duplex was tested by washing with 150 mM KCl. The duplex DNA content in the reactions of supernatant, 150 mM KCl wash, and the magnetic beads fraction was analyzed by agarose gel electrophoresis and staining with ethidium bromide.
f The synaptic complexes were formed with either homology or non-homology in the condition without or with spermidine as indicated. Following the challenge of AflIII digestion, the DNA species were revealed. Note that AMP-PNP nucleotide was used to assemble a stable RAD51 nucleoprotein filament.
Data are the mean ± s.e.m. from three independent experiments (n = 3). Raw data are provided as a Source Data file.

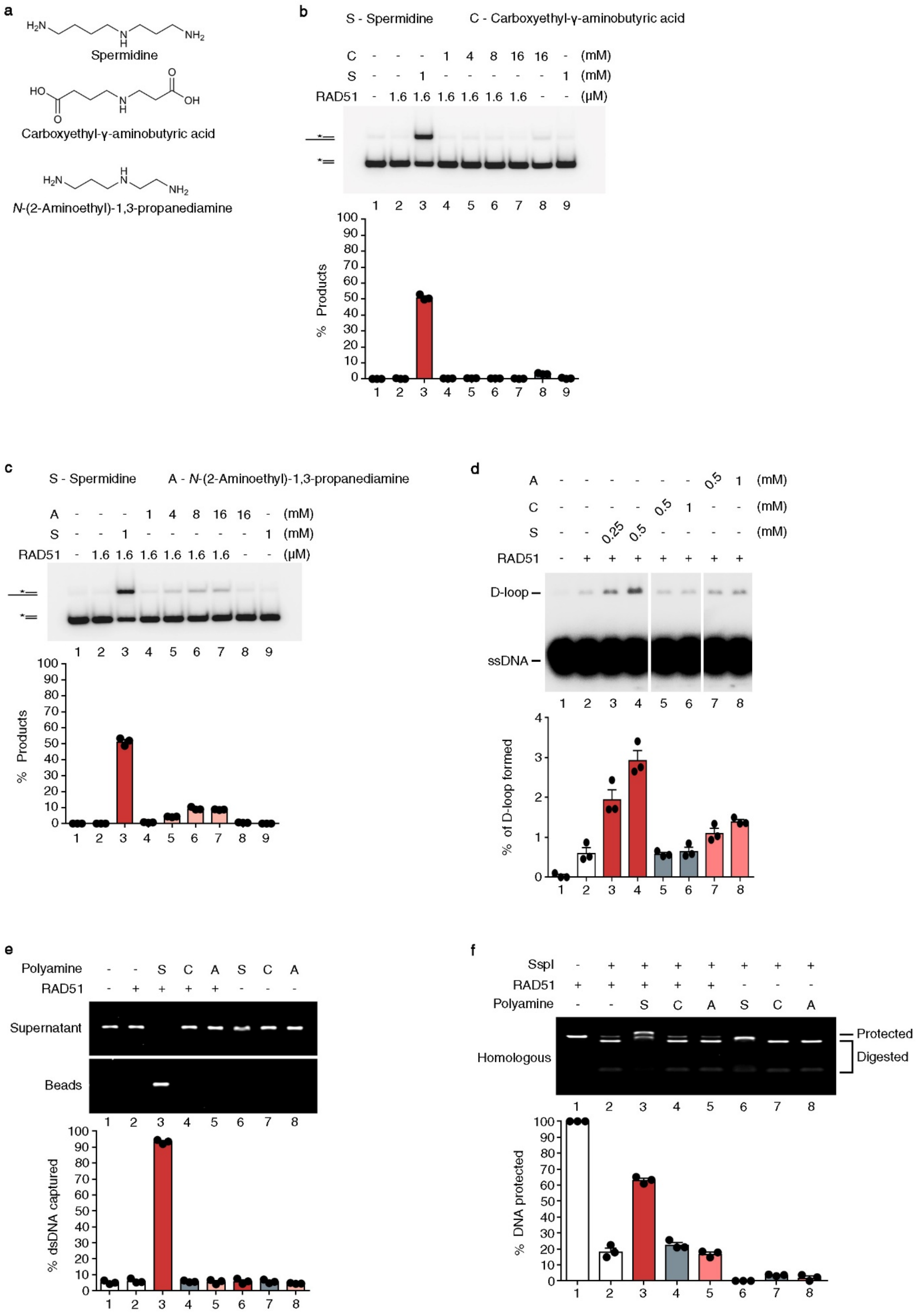

Supplementary Fig. 10 Synthetic polyamine analogs have no effect on RAD51 activity.
a Structures of spermidine and the two polyamine analogs.
b, **c** DNA strand exchange was examined without or with spermidine (S), carboxyethyl-γ-aminobutyric acid (C, structural analog of spermidine), or

N-(2-Aminoethyl)-1,3-propanediamine (A, ionic analog of spermidine). ATP was used as the cofactor. The reaction time was 20 min.
d D-loop formation was examined with the indicated concentration of spermidine (S) and two analogs (C & A).
e Duplex capture was examined without or with spermidine (S, 4 mM) or its two analogs (C & A, 4 mM) as indicated. ATP was used as the cofactor.
f The synaptic complexes were formed with homologous DNA substrate in the condition without or with spermidine (S, 2 mM) or two analogs (C & A, 2 mM) as indicated. Following the challenge of SspI digestion, the DNA species were revealed. Note that AMP-PNP nucleotide was used to assemble a stable RAD51 nucleoprotein filament.
b, c, d To normalize the percentage of product, each signal was subtracted from the percentage of product in control reaction (lane 1).
Data are the mean ± s.e.m. from three independent experiments (n = 3). Raw data are provided as a Source Data file.

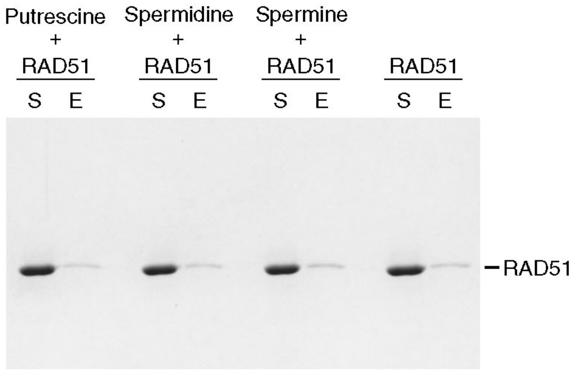

Supplementary Fig. 11 Polyamines do not interact with RAD51 directly.

Interaction between polyamines and RAD51 was examined by affinity pull-down assay. Biotinylated polyamines were immobilized on the streptavidin-coated magnetic beads and incubated with RAD51. The supernatant (S) and SDS eluate (E) from the pull-down reaction were analyzed by 10% SDS–PAGE and Coomassie Blue staining.

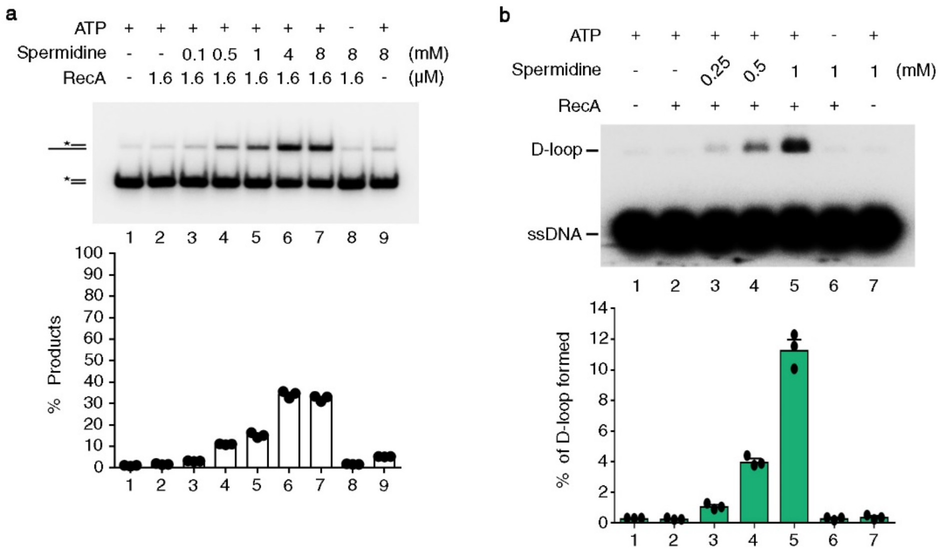

Supplementary Fig. 12 Spermidine stimulates RecA recombinase activity.

a Spermidine enhances the activity of RecA in DNA strand exchange in an ATP-dependent manner.

b D-loop formation was examined with the indicated concentration of spermidine.

Data are the mean ± s.e.m. from three independent experiments (n = 3). Raw data are provided as a Source Data file.

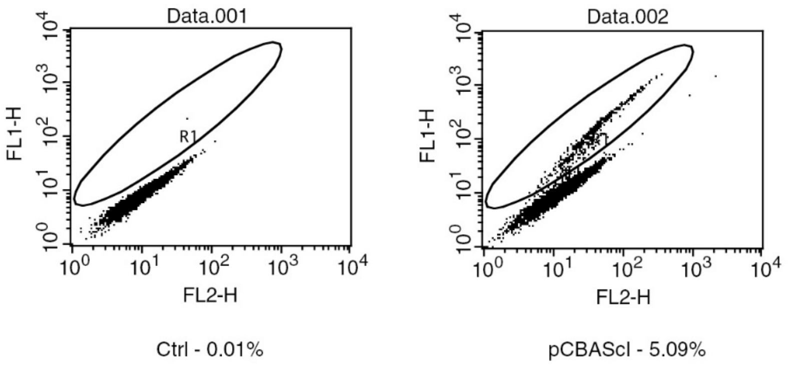

Supplementary Fig. 13 Gating strategy of I-SceI-based reporter assay.

Untransfected control cells (ctrl) were used to define the pattern of autofluorescence which was shown in FL1 and FL2 channels. Transfection with pCBASceI induced expression of GFP in cells. Thus, the proportion of GFP-positive cells was calculated by gating the FL1-positive cells compared to ctrl cells.

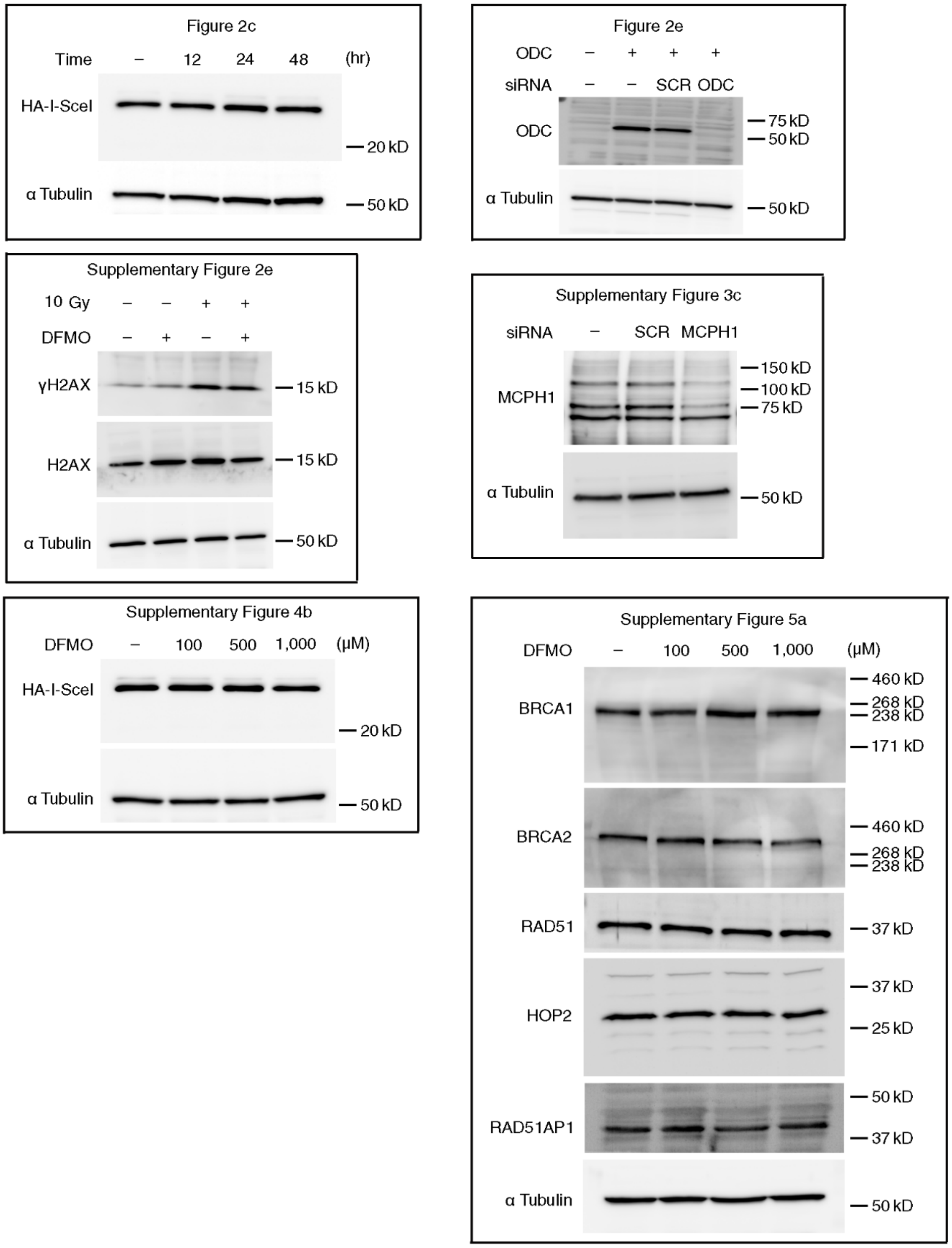

Supplementary Fig. 14 Uncropped protein immunoblots.
Original images of immunoblots shown in Figure 2c, e, Supplementary Figure 2e, Supplementary Figure 3c, Supplementary Figure 4b, and Supplementary Figure 5a.
